# Model-Driven Architectural Framework towards Safe and Secure Nuclear Power Reactors

**DOI:** 10.3390/s21155136

**Published:** 2021-07-29

**Authors:** Bassem Ouni, Christophe Aussagues, Saadia Dhouib, Chokri Mraidha

**Affiliations:** CEA LIST, University of Paris-Saclay, 91120 Palaiseau, France; Christophe.Aussagues@cea.fr (C.A.); Saadia.Dhouib@cea.fr (S.D.); Chokri.Mraidha@cea.fr (C.M.)

**Keywords:** architectural framework, Model-Driven Engineering, modeling language, Instrumentation and Control, nuclear reactor, safety and protection

## Abstract

Sensor-based digital systems for Instrumentation and Control (I&C) of nuclear reactors are quite complex in terms of architecture and functionalities. A high-level framework is highly required to pre-evaluate the system’s performance, check the consistency between different levels of abstraction and address the concerns of various stakeholders. In this work, we integrate the development process of I&C systems and the involvement of stakeholders within a model-driven methodology. The proposed approach introduces a new architectural framework that defines various concepts, allowing system implementations and encompassing different development phases, all actors, and system concerns. In addition, we define a new I&C Modeling Language (ICML) and a set of methodological rules needed to build different architectural framework views. To illustrate this methodology, we extend the specific use of an open-source system engineering tool, named Eclipse Papyrus, to carry out many automation and verification steps at different levels of abstraction. The architectural framework modeling capabilities will be validated using a realistic use case system for the protection of nuclear reactors. The proposed framework is able to reduce the overall system development cost by improving links between different specification tasks and providing a high abstraction level of system components.

## 1. Introduction

With the increasing architectural and functional complexity of systems, designers faced challenges in terms of specification, architecture definition, integration, qualification and certification. Accordingly, they deployed a Document-Based System Engineering (DBSE) approach carrying out text-based specifications. However, the DBSE approach raised several issues related to project management, system development process, specifications, trace-ability, collaborative work and standards compliance [1]. To overcome these weaknesses, system designers adapt the Model-Driven Engineering approach, known as MDE [2]. MDE attracted a lot of research interest in recent decades due to its ability to overcome the complexity challenges of systems. Moreover, MDE-based approaches allow analyzing the behavior, implementing and checking the safety of these systems where models act as the main development feature. The MDE area covers various standardized modeling languages, such as the Unified Modeling Language (UML) [3], and domain-specific languages (DSLs) [4]. DSLs can be based on the UML extension mechanism, called UML profiles, or on meta-models. Considering this, modeling stakeholders harness the capabilities of MDE to reduce a system’s complexity in many research fields and applications areas, including health [5,6], telecommunication [7], systems security [8], Internet of things [9,10], Robotics [11], Big Data [12,13], Aerospace applications [14], automotive systems [15], Cyber-Physical systems [16] and Climate change [17]. MDE languages allow the modeling of various constituents of systems, including the structure and the behavior. Thanks to model-based tools, the system performance can be pre-evaluated, and its design can be visualized at the early stages of development. Consequently, developers can reduce risks during the design phase with the early detection of errors. Moreover, models can ease the maintenance of systems during their operational phase [18].

In this work, we will focus on using MDE to reduce the development complexity of digital systems dedicated to safety and security of civil nuclear reactors, named Instrumentation and Control (I&C) systems.

In this context, several studies have been carried out that focus on the usage of MDE approaches and tools for safety and security of systems [19]. Accordingly, innovative model-based tools have assisted in the development of safety and security systems, taking into account their functional and non-functional requirements.

In [20], the authors explored the modeling issues that are currently being faced by a specific nuclear power plant monitoring system when managing regulatory safety requirements, standards and practices. They focused on studying requirements modeling techniques to understand their benefits and limits according to their company’s needs. The authors illustrated different facets of this problem by refining and analyzing the development process of I&C systems. The problematic has not been formalized, and the proposed approach is not generic and is only applied to their specific use-case. Furthermore, no architectural framework has been carried out.

The work in [21] focused on the user interface flexibility in model-driven engineering in order to scale up from simple applications to real case studies. The authors present three kinds of flexibility for improving the design and development of process models: the variability for equivalent choices, granular-ability for various abstraction levels and completeness for possibly optional and predefined reusable components. The authors validated their approach on a nuclear power plant use case.

Linnosmaa et al. [22] focused on the challenges pertaining to the overall safety of the I&C architectural design and, more specifically, the modeling and assessment of nuclear safety I&C systems at the architectural level. This paper describes the design process of early conceptual overall safety I&C architecture from a modeling point of view. Furthermore, the authors defined the requirements for a model-based approach to support the design and analysis of the design solution using the Architecture Analysis and Design Language (AADL). The authors reviewed the capabilities of this language for modeling overall safety I&C architectures and modeled a simplified example architecture of an APR-1400 nuclear power plant. In this work, the hardware architecture has not been refined. The researchers found that AADL is not adapted for modeling the overall I&C architecture due to the lack of analysis possibilities and standardized modeling approaches.

The authors in [23] addressed the question of formalizing the regulatory requirements for the nuclear domain expressing multiple different concerns, scatter and hinder the domain knowledge capitalization. They focused on improving the meta-model of tacit (non-written) requirements and practices. Accordingly, they proposed a dual Model-Driven Engineering (MDE) and Information Retrieval (IR) approach to address the nuclear regulatory requirements domain definition and assisted trace-ability based on the acquired requirements’ model. Their work focuses more on requirements but not on system components and their behavior.

Poirier et al. [24] presented a model-based engineering framework covering the I&C design process from the requirements to the design of I&C architectures. This framework is developed based on Increment and Papyrus open-source tools. It provides an extensible modeling environment as well as traceability and verification features. The physical components have not been considered. Compared with this work, our work will propose a new framework that is guided by an innovative approach covering all levels of abstraction and will be validated in a use case.

Cai et al. [25] compare two methodologies for designing interfaces of a primary cooling circuit of a pressurized water reactor (PWR). These methodologies are the Abstraction Hierarchy (AH) and Multi-Level Flow Modeling (MFM). They target to understand whether AH and MFM have provided a satisfactory answer. They conclude that AH and MFM are complementary in terms of their functions, and neither of them has provided a satisfactory answer to the foregoing question. The authors figure out the criteria for a more satisfactory answer, but the methodology to satisfy these criteria were to be considered in future work. We will compare our contribution with these two approaches in the results section.

Lin et al. [26] study the principles of interface design. They identify problems with Ecological Interface Design (EID) and proposed a new principle for human–machine interface design called Function Behavior State (FBS). They carried out a comparative study for EID and FBS on a simulated process plant system. We will compare our framework with FBS and EID in the results section.

Table 1 summarizes the advantages and disadvantages of the related work approaches. Various approaches missed the field problematic formalism, are not generic and are only applied to a specific use-case. In addition, no architectural framework has been designed for the modeling of I&C systems. In other works, the hardware architecture has not been refined. Other researchers found that the used language (for instance AADL) is not adapted for modeling the overall I&C architecture due to the lack of analysis possibilities and standardized modeling approaches.

Other approaches used languages that have their own graphical model and symbols. These graphical models are neither customizable nor extensible. They focus more on requirements but not on the system’s physical components and their behavior. Furthermore, some approaches consider only application tasks, activities, processes or events and do not fulfill a complete design or specification of tasks or events that the user needs to do.

To overcome these challenges, we propose a new collaborative methodology to model the I&C systems and communication between them at different levels of abstraction and check the consistency between them. The methodology is customizable and extensible. These models are used for I&C system design, performance evaluation, verification and validation. The proposed architectural framework covers various concepts, allowing system implementation, and includes different development phases and system concerns. Furthermore, we proposed a new I&C modeling language, including the different modeling rules needed to build different architectural framework views.

The architecture of I&C devices dedicated to the control and protection of nuclear reactors is quite complex. To overcome this issue, in this work, we aim to significantly reduce the development costs of these systems in terms of engineering time and equipment aspects. In order to address the cost improvement objectives of the engineering part, a new MDE-based methodology is proposed. To validate this approach, we extend the specific use of the open-source system engineering tool base, Papyrus [27], for the control and protection of nuclear reactors. The integration of this methodology within this tool encapsulates the main stages of use-case system engineering and carries out many automation steps and consistency checks between different design stages. In addition, it also improves the links between different tasks during the specification, design and development of the system.

The rest of this paper is organized as follows: The next section introduces a detailed description of the Model-Driven methodology for I&C systems engineering. Section 3 refines the Instrumentation and Control modeling language (ICML). Then, Section 4 illustrates the proposed approach on a nuclear reactor protection system. Finally, the conclusion and future works are drawn in the last section.

## 2. Model-Driven Methodology for I&C Systems

### 2.1. Architecture Framework for I&C Systems

Before defining a methodology to design and develop complex systems efficiently, it is mandatory to set up a coherent modeling framework that defines various concepts, allowing the design and implementation. This framework encompasses different development phases, all actors and various system concerns. This approach, called “architectural framework”, was standardized in [28], it is defined as “the set of conventions, principles and practices for the description of architectures established in a specific application domain and/or a community of stakeholders”. Key concepts of refining systems and their architectures are defined below as context for understanding the practice of architecture description:

- Stakeholders: an individual, team or organization that has concerns about the considered system in relation to its environment. A concern may be held by one or more stakeholders.
- Concerns: Throughout the life cycle of the system, concerns arise from system needs and requirements, design choices, implementation and operation considerations.
- Architecture view and viewpoints: an architecture description includes one or more architecture views. An architectural view addresses one or more concerns of system stakeholders. It expresses the architecture of the system of interest in accordance with a viewpoint. A viewpoint has two aspects: the concerns it expresses to stakeholders and the conventions it establishes on views. A view is governed by its viewpoint: the viewpoint establishes the conventions for constructing, interpreting and analyzing the view to address the concerns expressed by that viewpoint. Viewpoint conventions can include languages, notations, model types, design rules and/or modeling methods, analysis techniques etc.
- Architecture models: an architecture view is made up of one or more architectural models. An architectural model uses the modeling conventions appropriate to the concerns to be addressed [29]. These conventions are specified by the Model Kind governing this model. In an architectural description, an architectural model can be part of one or more architectural views.
- Model Kind: all conventions for a type of modeling. For instance, a model kind could be data flow diagrams, class diagrams, Petri nets or state/transition diagrams, etc.

**Figure 1:**
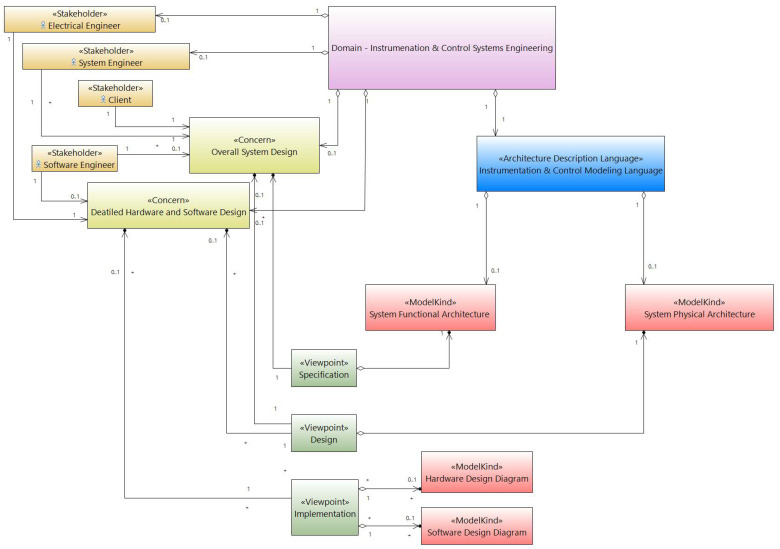
Class diagram of I&C systems Architectural Framework.

According to the ISO architectural framework working group [30], there is no architectural framework already established by a national or international community in the field of I&C of nuclear reactors. Then, following the architecture description standard, below, we define the basic concepts of the proposed I&C systems architectural framework, depicted in Figure 1.

- Stakeholders:
  - The client: The client provides a list of I&C functions to be performed and defines the associated specifications.
  - The system engineer: The system engineer is responsible for translating the functions to be implemented (and associated specifications) into a high-level architecture. The functions are translated to functional diagrams (FD), and this representation is independent of the implementation technology. This level is then refined, as the system development progresses, to Equipment Diagrams (ED), including technological details of implementation. The processing and functions at (FD) level are allocated to different (ED) level equipment of the system.
  - Software (SW) engineer: The software engineer can participate in collaboration with the system engineer in the development of (FD) functions. He is responsible for the application of system specifications to be carried out in the software/Hardware detailed design.
  - Hardware (HW) engineer: The HW engineer can participate in collaboration with the system engineer in the development of the system specifications. Furthermore, he is responsible, with the software engineer, for the application of system specifications in the software/hardware detailed design phase.
- The concerns:
  - Overall system design
  - Detailed hardware and software design
- The viewpoints:
  - Specification: This point of view establishes the conventions for the construction of architectural views, allowing the translation of customer specifications into system architectures regardless of technological implementation constraints.
  - Design: This point of view establishes the conventions for the construction of functional architectural views, taking into account technological implementation constraints.
  - Implementation: This point of view establishes the conventions for the construction of behavioral views, which contain all the information necessary for the manufacture of I&C cabinets.
- Model kinds:
  - System Functional Architecture: It describes the system functional entities and the relationships between them and their sub-functions without considering the technological implementation.
  - System Physical Architecture: It describes the hardware entities at a high level of abstraction and the relationship between them to represent the equipment behavior taking technological constraints into account.
  - Hardware Design Diagram: It refines the hardware devices of different system devices.
  - Software Design Diagram: It includes the software behavior and the relationship of different software applications running on various system devices.

### 2.2. I&C Modeling Language

The architectural framework presented in the previous section defines three viewpoints that establish the conventions for developing I&C architectural views. These views must be developed using a graphical modeling language, such as an Architecture Description Language, that will encapsulate all of the concepts and modeling rules needed to build these views. The proposed language is named the Instrumentation and Control modeling Language (ICML). The latter is the result of the aggregation of a domain-specific modeling language (DSML) [31], as shown in Figure 1.

**Figure 2:**
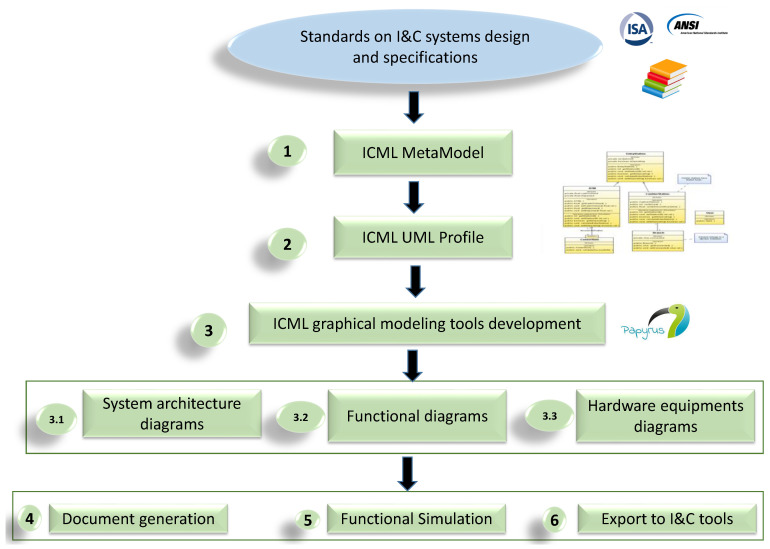
The ICML and associated model-based tools development process.

The development of ICML and the associated graphical editors follows the flow as depicted in Figure 2. Considering the I&C specification and the study of existing standards for I&C systems graphical notation [32], the ICML is designed based on these two syntaxes:

- An abstract syntax or Metamodel: The metamodel defines different concepts manipulated in the domain and structures the relationships between these concepts and their semantics into a coherent unit. UML is used to formalize these concepts and define the relationships between them. The concept’s semantics can be modeled textually or formally via a dedicated language, such as OCL (Object Constraint Language) [33].
- A concrete syntax: This defines the textual or graphical notations for the modeling language. The links between the abstract syntax concepts and the concrete syntax notations are also defined. Several concrete syntaxes can be defined for the same abstract syntax, which allows several representations for the same metamodel. To develop the ICML concrete syntax, the UML profile is used, which is to say, we define stereotypes extending the UML meta-classes. The major advantage of using the UML profiling approach is to benefit from a set of existing tools, both commercial and open-source.

Based on meta-models, we developed an architectural framework for I&C systems with the graphical editors. These model-based editors, which correspond to the model kinds in the architectural framework presented in Figure 1, are developed as supporting graphical tools for the ICML language. The modeling language tool used for developing the architectural model-based framework is Papyrus [27]. Papyrus is an open-source model-driven engineering tool and available in the Eclipse platform [34]. Various modeling features of Eclipse Papyrus are designed to be customized and to maximize reuse. Therefore, we adapt this modeling environment to shape the proposed ICML language, taking into account I&C systems development methodology and requirements.

The developed framework allows modeling I&C devices at system, functional and physical levels. The ICML models will be used to achieve the simulation of system functional behavior, export data to other I&C tools and generate dedicated documentation.

### 2.3. Architectural-Based Methodology Formalism

As explained in the previous section, the architectural framework encompasses various system development phases. The refinement of these phases is mandatory to enrich the ICML language to cover various concepts of I&C in civil nuclear power plants. For this reason, we divide the I&C systems development process into steps and involve the participation of stakeholders throughout this process within an architecture-based methodology.

The I&C systems engineering process involves several steps, each of which has impact on the next one.

**Figure 3:**
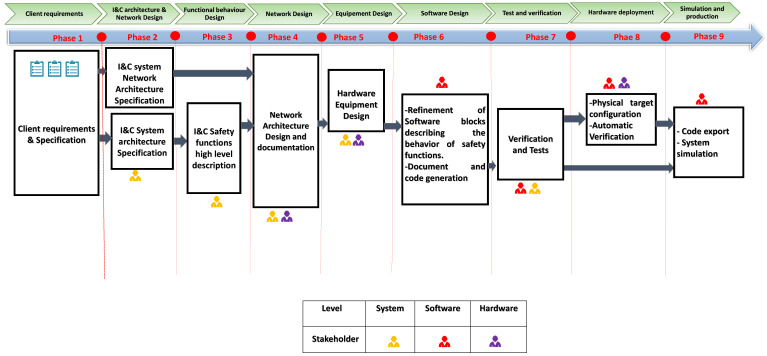
Methodological flow of the I&C systems development process.

The first step consists of defining the system architecture diagrams that abstract the overall outline of this system and the inter-operability between its components. Furthermore, experts define the set of the inputs/outputs of the overall system, taking into account several naming rules. Then, the functional behavior of the system is defined; it is described as a sequence of functions, their sub-functions and their interactions with whatever their technological implementation. This functional level, named Functional Diagram (FD) level, takes the form of various diagrams including these functions. Finally, designers define the Equipment diagram (ED) level, where the allocation of system functions on the hardware platform is detailed. Figure 3 depicts different steps of the I&C systems development process. Different phases are detailed below:

- Phase 1—Client Requirements: In this step, documents expressing customer needs in terms of safety requirements and functions (from the overall I&C systems architecture) are defined. These documents, provided in mainly document formats (Word and Excel, etc..), are the main inputs of the system architecture specification (phase 2) and will also serve as the functional design (phase 3).
- Phase 2—I&C Architecture and Network Design: In this phase, the specifications of the I&C system network architecture and global architecture are defined, taking into account the customer specifications. The global architecture is then used for the I&C safety functions high-level description in Phase 3.
- Phase 3—Functional behavior design: This describes the system as an arrangement of functions, their sub-functions and their interactions, with the abstraction of technological implementation, of which FDs represent the behavior. It is a customer exchange medium. For this behavior definition, various files of global architecture in Phase 2 are used.
- Phase 4—Network design: In this step, designers define the way the system equipment are structured to efficiently transit data between different system blocks. Furthermore, the network design takes into account the functional behavior and the physical architecture to define system input and output data and how the data are transferred over the I&C system.
- Phase 5—Equipment design: During this phase, the physical devices are designed considering the network architecture and are used for the generation of hardware documentation as well as for the software design in Phase 6.
- Phase 6—Software design: The behavior of various equipment is manually developed and integrated into a design environment for model-based design, simulation, verification and code generation.
- Phase 7—This phase consists of system tests & verification. Furthermore, documentation verification and functional tests are carried out. The outcome of this last step will be used for modeling physical architecture, simulation and code generation.
- Phase 8—Hardware devices configuration and deployment: Using specific software tools, experts generate executable binaries on target boards for production and simulation purposes.
- Phase 9—Simulation and production: This step consists of running simulations and codes on the hardware platforms, as well as the documentation of the I&C system.

Throughout the methodological flow, stakeholders are involved to develop, validate or verify the various phases of development process.

### 2.4. Proposed Approach

**Figure 4:**
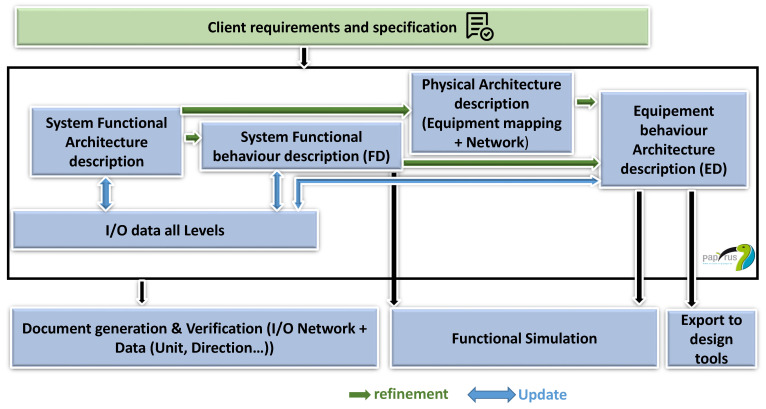
The ICML-based model-driven approach.

Based on ICML, a new model-driven approach is proposed and integrated within the model-based framework, as shown in Figure 4. This methodology aims to reduce the development cost of I&C systems and automate and facilitate the consistency checks at different levels of abstraction. The Papyrus-based system engineering approach will replace a part of the existing process described in the last section, where optimization in terms of resources has been identified. The first step of the approach consists of the description of the system functional architecture and the refinement of its behavior as a sequence of functions, their sub-functions and their interactions. The physical architecture is described subsequently as hardware equipment blocks and the network architecture is specified. After that, the system’s physical behavior and the allocation of its functions on the hardware platform are detailed. Throughout different phases of this approach, a database of I/O, including data at different levels, is updated.

The developed models are exploited for functional simulation, document generation and exports to other tools.

**Figure 5:**
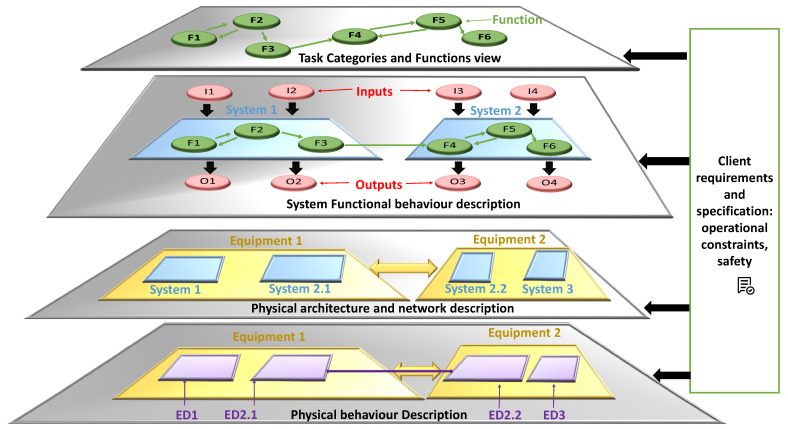
The different levels of abstraction of the proposed I&C framework.

Figure 5 details different levels of abstraction of the proposed framework. The first level of design covers the expected functions and their classification according to their safety category. Then, the behavior of these functions is refined: each system is an arrangement of functions, their sub-functions and their interactions with whatever their technological implementation. Furthermore, the system I/O data are modeled, considering the naming conventions, during this stage. For instance, in the example of Figure 5, we have three systems: system 1, system 2 and system 3. System 3 is not represented at the functional behavior description level for space reasons. The third level of abstraction defines the allocation of system functions on the hardware platform. The latter is modeled as a set of equipment. A system can be mapped on two different equipment: for example, System 2 is implemented on two equipment: the sub-systems system2.1 and system2.2 are mapped, respectively, in Equipment1 and Equipment2.

Finally, the detailed specification and equipment behavior (ED) are modelled, taking into account the technological implementation (failure processing, data definition for the monitoring system). A system’s ED can describe the behavior of two different equipment. In Figure 5, ED2.1 and ED2.2, respectively, represent the behavior of Equipment1 and Equipment2, implementing System 2.

## 3. ICML Language Description

### 3.1. Functional Level

At the functional level, the basic element of the functional architectural description in the I&C development project is the safety function, such as the “Reactor Trip” or the “Neutron Flux measurement”. It is characterized by a name, a safety category and possibly a response time. A safety function can itself be divided into safety sub-functions. For instance, the “Reactor Trip” function can be broken down into the “Power of PCP or voltage low” sub function, “Pressure of primary circuit high,” etc. A safety function can be reused identically in many projects. It is possible to include it in a library and instantiate it in a particular system.

The second basic element at this level is the system performing one or more safety functions. It is characterized by a name, a safety class and its functions. A system can be reused identically from one project to another. For this reason, it will be encapsulated within a library to instantiate it later in a particular project. Other elements of the functional architectural description are the sets of sensors and actuators describing the system means of access to the environment, i.e., to the system Inputs/Outputs. Each element is characterized by its name, its safety class and the list of using systems.

Besides, Input/Output signals constitute a core component of the functional architectural description. They are referred to as functional signals. Each signal is characterized by a name, a type (binary or analog), a direction (input or output) and a list of associated systems. Indeed, these signals are transmitted from one system to another via logical communication links. A logical communication link between two systems is characterized by a name, a transmitter system, a receiver system and a list of associated functional signals.

### 3.2. Physical Level

The physical architectural description of I&C systems incorporates standard implementation elements:

- Electronic computational and input/output boards: Each board is characterized by a name and a type. A board can be reused identically from one project to another, and it will be integrated in a library and instantiated in a particular rack.
- The racks: Each rack is characterized by a name, a usage and a list of owned boards. A rack can be reused identically from one project to another, and it will be encapsulated in a library and instantiated in a particular cabinet.
- The cabinets: Each cabinet is characterized by a name and a potential list of contained racks. It will also be included in a specific library and instantiated in a particular equipment.
- Equipment is a basic physical component of systems. This equipment may or may not be made up of cabinets. Each equipment is characterized by a name, a list of implemented functions, a safety class, a type of technology, a potential set of cabinets, a set of implemented systems and a list of manipulated signals (entry signal, output signal or exchange with other equipment).
- Physical signals are the signals exchanged between equipment. They are used to refine functional I/O signals and ensure the physical implementation of the system. A physical signal is characterized by many attributes, such as the type and direction (as for functional signals), the customer code, the code related to implantation technology, etc.

### 3.3. Meta-Models and UML Profiles

#### 3.3.1. System Architecture Profile

**Figure 6:**
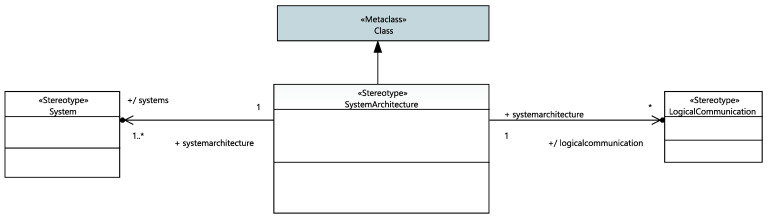
System Architecture profile.

The project is modeled as a stereotypical UML class named “System Architecture”. As shown in Figure 6, it owns a structural diagram to visualize its systems, sensors and actuators. Furthermore, this class has one or more structural diagrams to represent inter-system data exchange using sensors and actuators. The proposed framework will automatically update the list of systems and the list of logical inter-system communication links needed to build the project.

#### 3.3.2. Safety Functions and Systems

**Figure 7:**
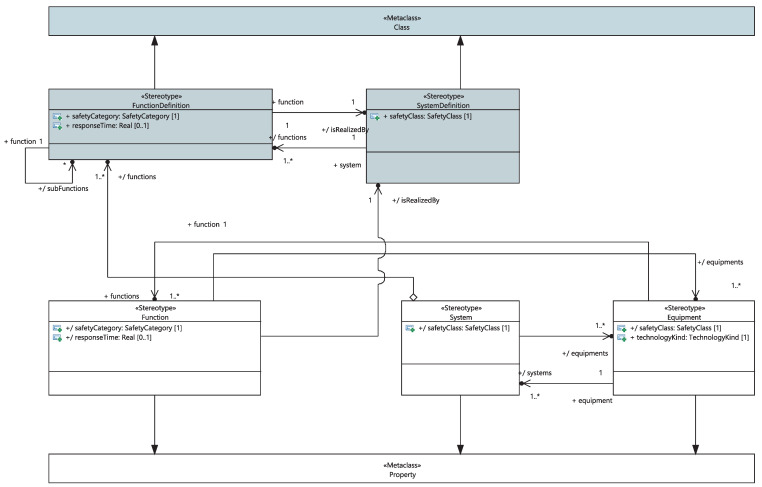
Meta-model of functions and systems.

The development methodology begins with the definition of safety functions “FunctionDefinition” and system “SystemDefinition”, as stereotypical UML classes, for which the system engineer provides the list of functions “Functions” they realize, as shown in Figure 7. Furthermore, the “SystemDefinition” has as attribute a stereotypical UML “Function” class and is typed by its corresponding “FunctionDefinition”.

Using the functional decomposition relationships, the tool calculates accordingly for each “FunctionDefinition”, the list of its potential sub-functions “subFunctions” and the system “SystemDefinition” that carries it out.

**Figure 8:**
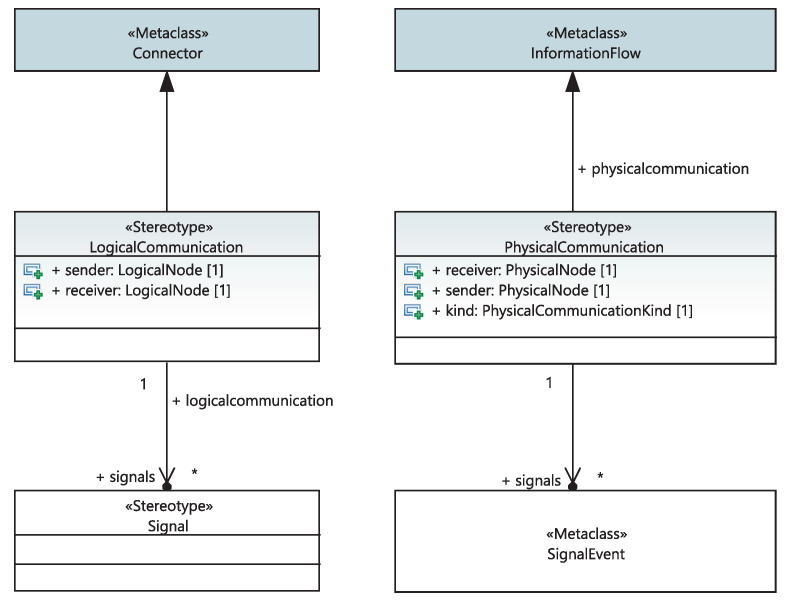
Meta-model of logical and physical communications.

**Figure 9:**
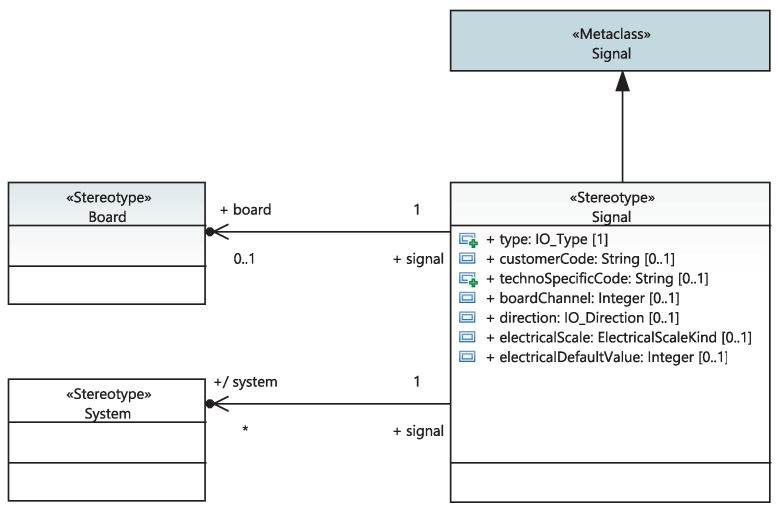
Meta-model of functional and physical signals.

When the system engineer describes, via a structural diagram, the composition of their “SystemDefinition” into functions placed in the racks (“Function”), the “safetyCategory”, “responseTime” and “isRealizedBy” attributes of these functions are automatically updated. Moreover, high capabilities of the proposed framework will allow saving time to different stakeholders. Specifically, When the system engineer describes the composition of their project into systems, the “safetyClass” attribute, the set of functions realizing the system and the list of its equipment are automatically updated. The list of equipment that implements it is updated when the system engineer sets the list of equipment “Functions”. The system engineer is also required to specify the list of (I/O) signals of the system. Furthermore, the logical communication links between systems are modeled as extended UML connector-linking systems placed in racks. As depicted in Figure 8, each link is stereotyped as a “LogicalCommunication” class characterized by the “sender” and “receiver” attributes and the list of signals transiting from the transmitter to the receiver. An I/O signal is modeled as a stereotypical UML signal with a type and direction at two levels of abstraction: the physical and system levels. The System I/O signals are refined to physical I/O signals. Both I/O signals are modeled using the same UML stereotype. As shown in Figure 9, functional I/O signals are associated with a list of systems, as they can be transmitted through logical communication links between systems. On the other hand, physical I/O physical signals can be associated with an electronic I/O board.

#### 3.3.3. Physical Equipment

**Figure 10:**
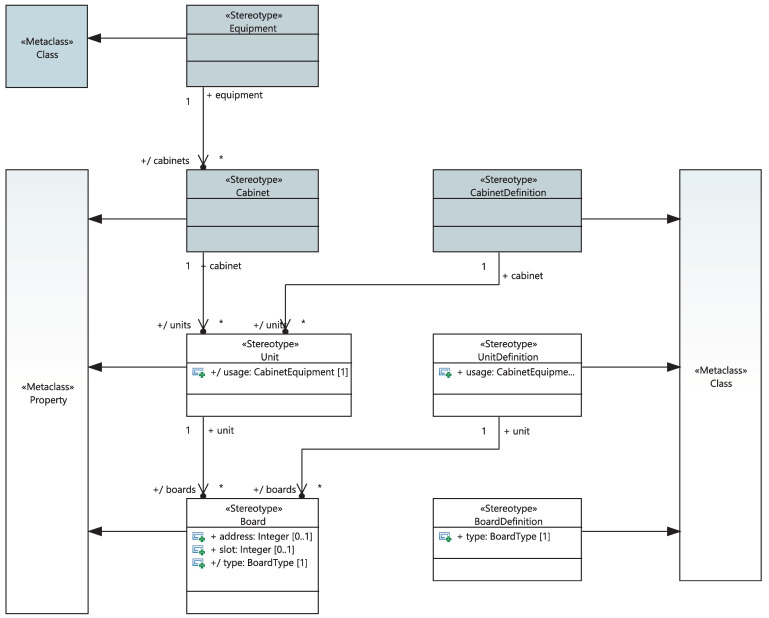
Meta-model of the physical equipment.

I&C equipment can be either a compact unit or a device composed of boards, racks, or cabinets. In the latter case, we define the set of boards “BoardDefinition” on the racks, the list of racks “UnitDefinition” on cabinets, and different cabinets “CabinetDefinition” of the system. As shown in Figure 10, the “BoardDefinition” is a stereotypical UML class defining the board and its type. The stereotyped class “UnitDefinition” (1) has different “Board”s which are “BoardDefinition”-typed, (2) has a special use in a cabinet defined by the attribute “usage” and (3) will be automatically updated if the user changes the properties of its boards. The stereotyped class “CabinetDefinition”, in turn, includes various “Unit”s having a “UnitDefinition” and will be also automatically updated in case the included rack’s properties are changed.

**Figure 11:**
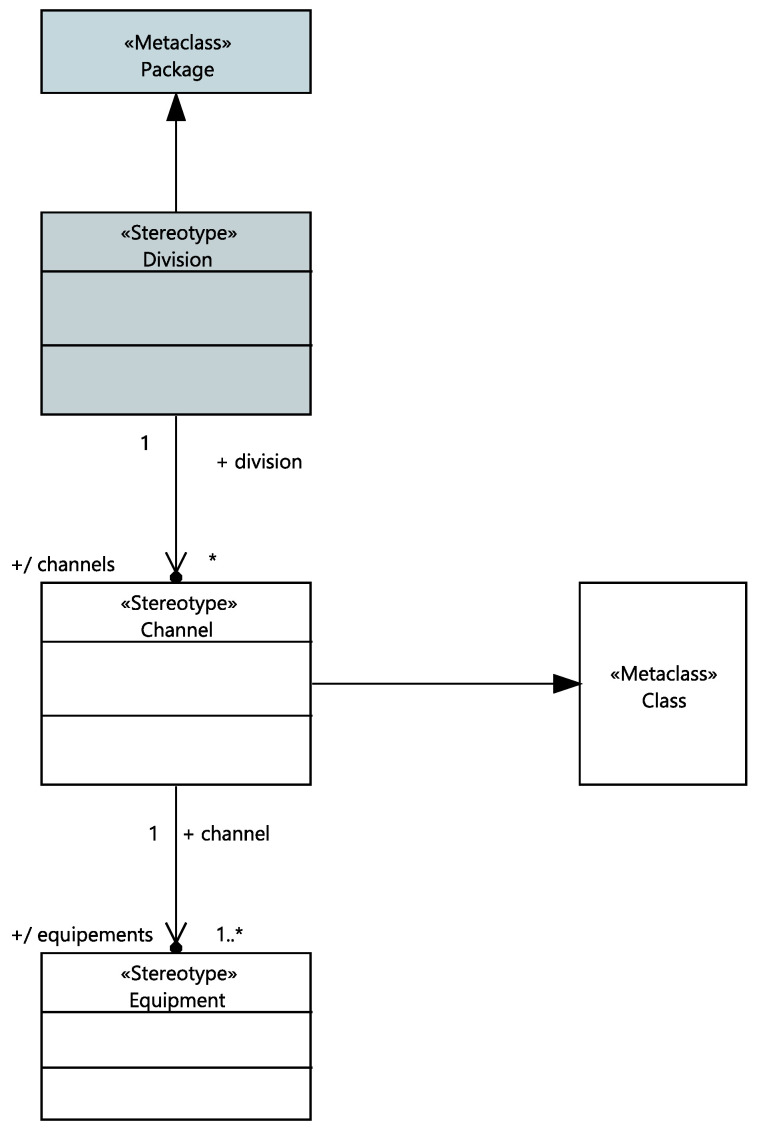
Meta-model of divisions and Channels.

The stereotypical UML class “Equipment” is characterized by its associated functions, systems and its technology kind. It includes potential “Cabinet”s. The Equipment’s safety class, list of its potential cabinets and associated systems are updated automatically. Figure 11 depicts the stereotyped package “Division” has a list of automatically updated associated channels (“Channel”). A Channel is a stereotypical UML class with a list of equipment associated with.

### 3.4. Views

As explained in Section 2.2, the ICML architectural framework provides three viewpoints: Specification, Design and Implementation. These viewpoints allow the system engineer to describe the functional behavior of the I&C system architecture and its physical architecture as separate blocks: systems and equipment. During the physical description, the system engineer will be assisted by client requirements, the hardware electrical and software engineers. At the system functional level of the ICML framework, the following concepts are considered:

- Safety function library package: it contains “FunctionDefinition”.
- System library package: it contains many systems, such as “SystemDefinition”. These systems define various functions “FunctionDefintion” as a stereotypical property “Function”.
- The System Architecture class: it includes the various sub-systems of the I&C system; they are represented in a stereotypical property named “System”, which will include different “SystemDefintion”s.
- The Signal library package: it contains many “Signal”s.

Consequently, to model the project class diagrams at this functional level, the proposed tool will allow creating: a Package, a Function Definition, a function, a System Definition, a system, and a signal. At the system physical description level, the following concepts are considered:

- Division package: this contains Channels or Equipment.
- Board library package: this includes a set of “BoardDefinition”s.
- Unit library package: this contains many “UnitDefinition”s, with various “BoardDefinition”s as stereotypical property “Board”.
- Cabinet library package: this contains various “CabinetDefinition”s with “UnitDefinition”s as stereotypical property “Unit”.
- Equipment class: this includes many “CabinetDefinition”s as a stereotypical property “Cabinet”.
- Channel class: this includes “Equipment” modeled as classes.

Hence, at this physical level, the palette of the proposed tool will allow creating a package, a division, a channel, equipment, a Cabinet Definition, a Cabinet, a Unit Definition, a Unit, a Board Definition, a board, and a signal.

## 4. Use Case: Reactor Protection System

**Figure 12:**
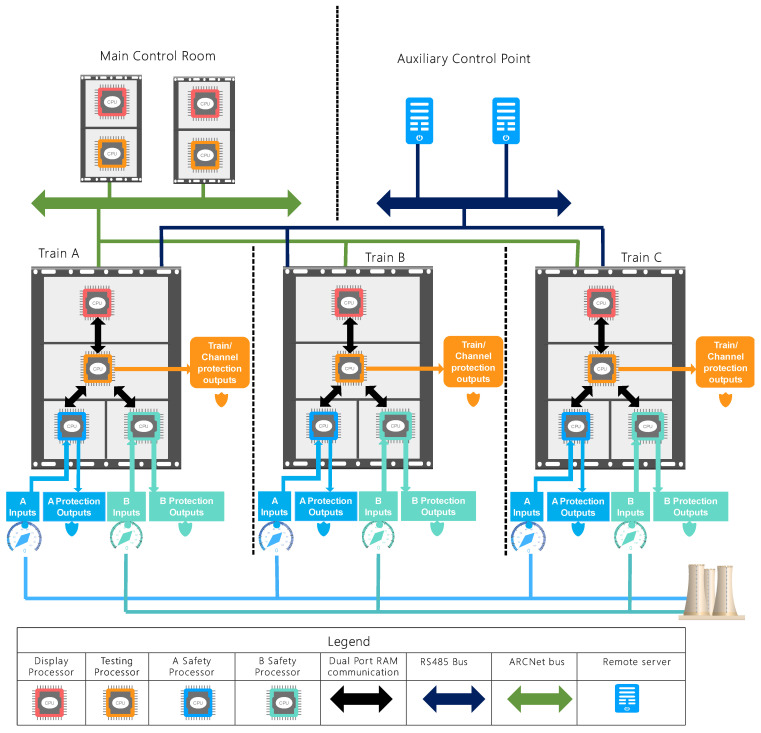
An overview of the used case RPS.

The used case system is a Reactor Protection System (RPS) gathering various safety and security sensor-based devices embedded in a civil nuclear power plant aiming to safely shut down the civil nuclear reactor if radioactive materials leak. The (RPS) is composed of various safety functions placed on different hardware devices. The use case digital (RPS) is designed by experts from the Tsinghua University of China and described thoroughly in [35]. It is based on electronic boards, sensors, actuators, computers and software tools that are deployed within various sub-systems to ensure the safety and security of a 10 MW high-temperature gas-cooled experimental reactor (HTR-10). Figure 12 depicts the architecture of this RPS, and it is composed of three parts. The first part consists of redundant protection logic units in three channels (trains); the second part gathers the surveillance stations and Post Accident Monitoring System located in the main control room; the third part is the monitoring system located in the auxiliary shutdown point. The system outputs are the protection signals resulting from a hard-wired 2/3 voting logic operation of the triggering signals in three redundant channels. The design basis accidents and corresponding protection variables used in the HTR-10 nuclear reactor are refined in [35]. The RPS outputs a set of warnings and protection actions for each protection variable. For better safety management, developers divided the protection variables into two groups A and B. In each group, at least one protection variable is set up to protect each initial event, and each group is independently implemented in different processors to act as diversity within each channel. These protection actions are classified into four classes: Protection A (PA) includes the generation of an emergency trip signal to drop the control rods, trip the helium blower and isolate the secondary loop. The protection action B (PB) is referred to the relief of the steam generator. The protection action C (PC) consists of the isolation of the fuel loading/unloading system and the helium purification system from the primary loop. Finally, the protection action D (PD) targets isolating the thermal measurement system from the primary loop.

The system surveillance stations collect data from the channel station using ARCNET communication protocol dedicated to local area networks. The auxiliary Control system gathers information from channel stations through 2 hardwired cables ensuring an RS485 serialized communication. Each channel consists of four processor-based boards dedicated to data display, testing and checking the safety. Considering the input variables, the A Safety processor (AIPC) and the B Safety processor (BIPC) run different safety operations related, respectively, to two groups of protection variables A and B and generates the corresponding protection actions. The testing processor (TIPC) serves to monitor the status and checks the outputs of AIPC and BIPC. Using a dual-port RAM, it will forward data about itself, the (AIPC) and (BIPC) to the display processor (SIPC). In addition, it communicates these data to both the system surveillance stations via the ARCNet network and the monitoring systems in auxiliary shutdown point via RS485. Therefore, (SIPC) only displays data about the channel status on screens upon the request of relevant stakeholders.

### 4.1. Functional Level Modeling

**Figure 13:**
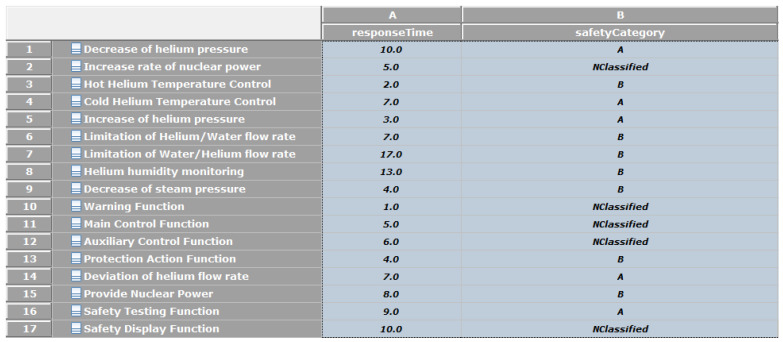
Tabular representation of the safety functions.

A package called “SafetyFunctionLibrary” is modeled to define various safety functions; the modeled library has three graphical representations: tree, tabular or class diagram representations. Figure 13 shows the tabular representation of these safety functions. A safety function can include many sub-functions.

**Figure 14:**
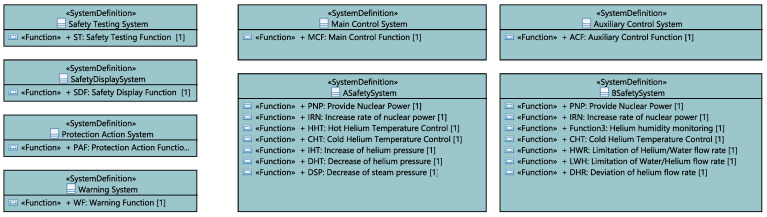
RPS systems.

After that, we define the set of systems following the proposed ICML language development flow. For the RPS case, we define eight systems on racks within the same “SystemLibrary” package, as depicted in Figure 14. Each system is placed on a rack and runs one or more safety functions.

**Figure 15:**
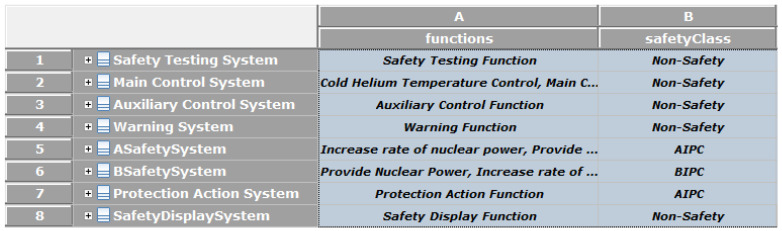
Safety class and associated safety features of systems on the racks.

The system is characterized by two attributes. The first attribute is the system’s safety class entered by the system engineer while its functions are automatically updated according to system structural diagram. The second attribute consists of these functions’ set. Then, as depicted in Figure 15, the ICML framework generates a table showing different systems placed on racks.

**Figure 16:**
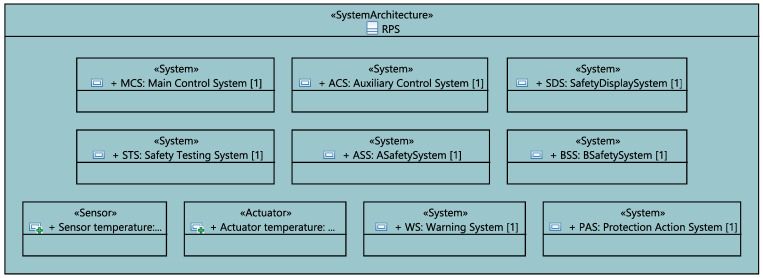
RPS sub-systems, sensors and actuators.

At a higher level of abstraction, the RPS project is modeled as a class with a structural diagram describing its different systems, sensors and actuators, as shown in Figure 16.

**Figure 17:**
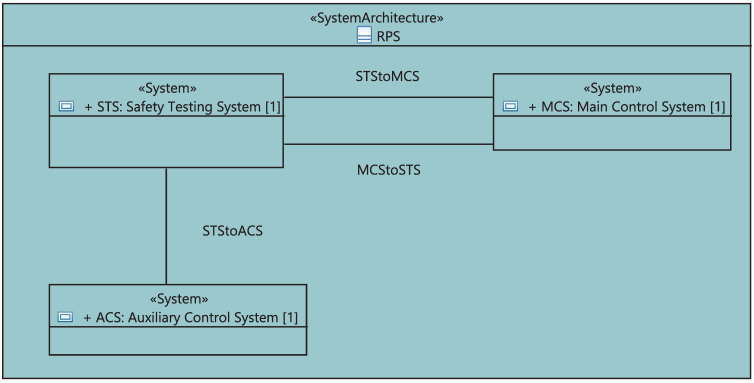
Communications between the RPS’ sub-systems.

The input/outputs of all systems are represented as stereotypical “Sensor” or “Actuator” classes. Their assignment to systems can be edited using an attribute. Communications between systems are represented by links specifying the nature of communication, as depicted by Figure 17. Furthermore, each functional signal is characterized by a set of attributes to specify its type, binary or analog, whether it is an input or output, its I/O board, etc.

### 4.2. Physical Level

**Figure 18:**
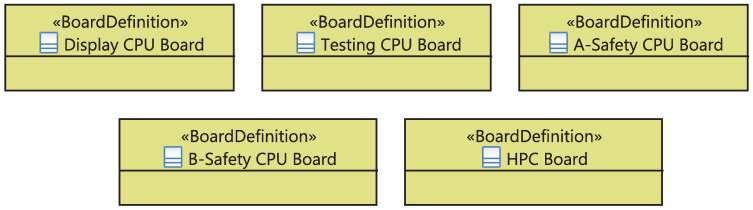
Electronic boards on the rack.

**Figure 19:**
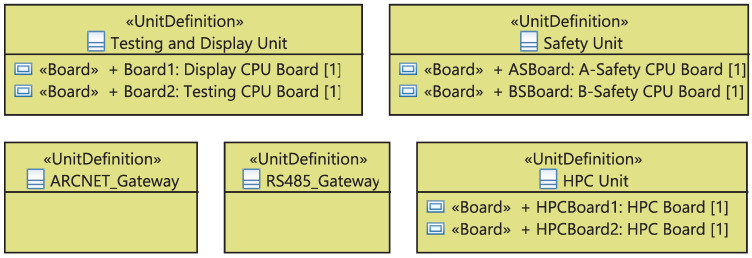
Units on the rack.

At the physical level, the ICML framework allows the definition of a package gathering five kinds of electronic boards placed on racks, as showed in Figure 18. Each board has a particular type, specified by the attribute “type”, characterizing its hardware architecture. Furthermore, another package is modeled to define the four types of used racks, as depicted in Figure 19. Each Rack/Unit has a particular type and a specific configuration of electronic boards.

**Figure 20:**
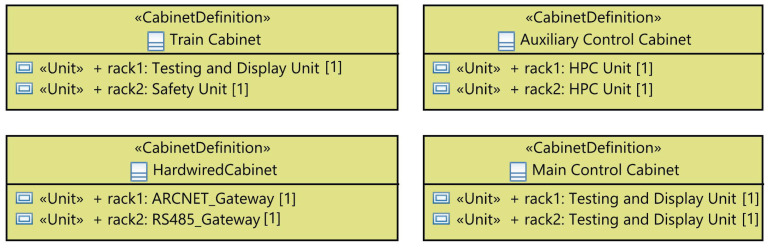
RPS cabinets.

Furthermore, we define the set of cabinets used for this system. The modeled package for this purpose defines four types of cabinets gathering various units and racks required to model the system, as depicted in Figure 20. Each cabinet has a particular type and composition; for instance, the Train cabinet gathers the units required to carry out safety, testing and display functions.

**Figure 21:**
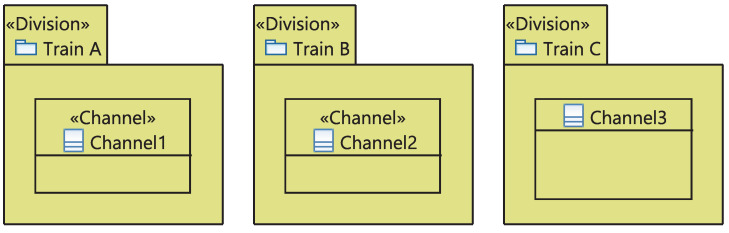
Divisions and channels.

Furthermore, the equipment of (RPS) system are defined in a package gathering the set of divisions and channels, as shown in Figure 21. The use case system has three divisions, “Train A”, “Train A” and “Train C”, with one channel each as explained above in the description of the RPS system.

**Figure 22:**
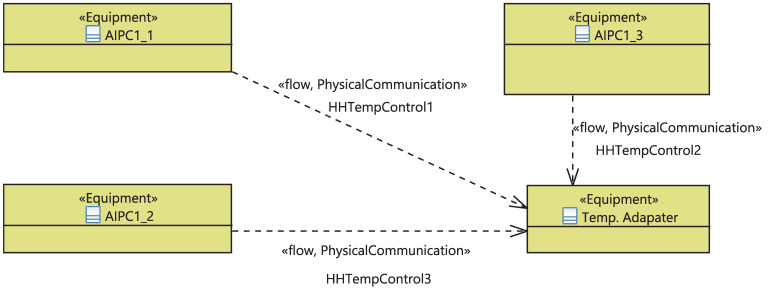
Communication links between the equipment implementing different functions.

Moreover, communications between the equipment realizing the same functions or different functions are modeled at this stage. Figure 22 highlights the communication links between the equipment implementing different functions, which are the cores of the APIC and a temperature adapter device, through oriented communication links holding physical signals.

**Figure 23:**
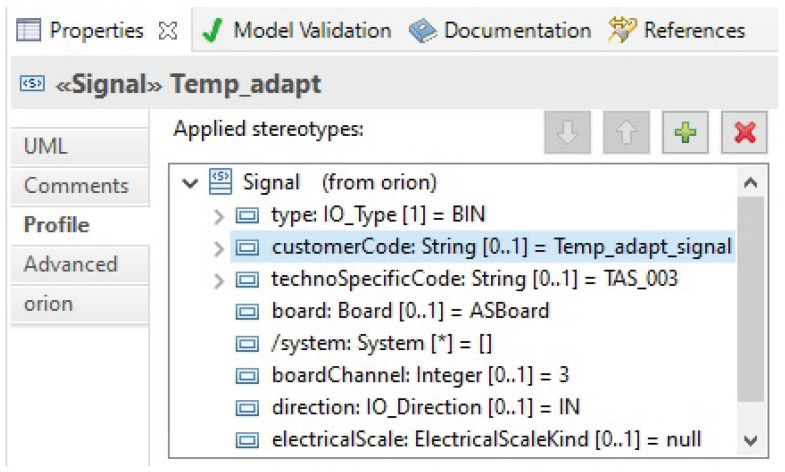
“Temp_adapt” physical signal attributes.

Physical signals are modeled using the ICML framework within the equipment holding them. Thus, for example, as shown in Figure 23, “Temp_adapt” is a signal characterized by various attributes, such as its holding board, channel, holding system, direction, etc.

### 4.3. User-Perspective Optimizations

The MDE solution is interdisciplinary and collaborative and allows an iterative design so that various system parts (functions, equipment, inputs/outputs, etc.) can be enriched, modified or updated throughout the design process. Moreover, the framework is synchronized in a way that different views are linked together; every change in a view causes an update in the other one accordingly.

The framework reduces the design time as it maximizes the tasks’ automation, improves communication between different stakeholders during the specification, design and development of the system, increases system reliability through automatic checks and facilitates corrections and changes being designed. To support this, The tool removes various manual tasks such as refilling EDs with redundant data already modeled in FDs. It checks the consistency of I/Os at each phase, according to naming rules.

### 4.4. Comparison with Other Approaches

In [25], the Multilevel Flow Modeling (MFM) has its own graphical model and symbols and does not address how to visualize information. However, every part of the Papyrus-based framework may be customized: the UML profile, the model explorer, the diagram notation and style, the properties views, the palette and creation menus. Consequently, in our approach, each functional or equipment component (operations, links, etc.) graphical representation has been fully customized and is visualized and meets specific graphical requirements. Furthermore, MFM does explicitly represent the knowledge of principles that govern the operation of a dynamic system. However, our functional and equipment models have a dynamic representation so that the user can update the kind of each component and the graphical interface and the behavior of this component change dynamically. On the other hand, the Abstraction Hierarchy (AH) concerns the domain: it models the system with no consideration of specific application tasks, activities, processes or events. The authors showed that AH does not fulfill a complete design or specification of tasks or events the user needs to do, whereas, in our proposed approach, we focus on the extended UML profiles to model the complete functional, architectural and equipment components required in the specification and refines its properties and characteristics.

According to [26], the display of the various EID levels of information on the same front can make the operator distracted by the information that does not contribute to solving problems at hand. Furthermore, EID framework information may be overlapped so that different levels of information are displayed without concerning their syntactical issues. Furthermore, there is generally a lack of consideration of time constraints. The proposed Function Behavior State (FBS) framework is only focusing on modeling particular aspects such as the user’s role, values and needs, as well as producing an explicit representation of failures and redundant functions. The hardware architecture is not considered in this work.

## 5. Conclusions and Perspectives

In this paper, we proposed a new interdisciplinary collaborative methodology to model at different levels of abstraction different I&C components and communication between them. These models are used for I&C system design, performance evaluation, verification and validation. The proposed ICML architectural framework defines various concepts, allowing system implementations and includes different development phases and system concerns. In addition, we defined a new I&C modeling Language (ICML), including different modeling rules needed to build different architectural framework views. Future works will focus on the enhancement of the synchronization between components at different levels of abstraction, the integration of simulation within the proposed framework to execute UML models, provide control, observation and animation facilities over these executions.

## Figures and Tables

**Table 1:** Related works comparison

Approach	Pros	Cons
Linnosmaa et al. [22]	- Modeling and assessment of nuclear safety I&C systems at an architectural level	- A lack of analysis possibilities and standardized modeling approaches (AADL)
Ceret et al. [21]	- Scaled the user interface, variability for equivalent choices, granular- ability for various abstraction levels and completeness for possibly optional and predefined reusable components.	- Graphical interface is not extensible and not dynamic
Sannier et al. [23]	- Refined and improved the nuclear regulatory requirements domain	- Focused only on the requirements but not on the system components and their behavior.
Poirier et al. [24]	- An extensible modeling environment. - Traceability and verification features.	- The physical components were not considered - Not guided by an approach and not validated with a realistic use case.
MFM approach (Cai et al. [25])	- Modeled the processes	- Fixed and not extensible graphical representation. - No dynamic representation of system behavior
AH approach (Cai et al. [25])	- Modeled the domain	- No consideration of specific application tasks, activities, processes or events. - Did not fulfill a complete design or specification of tasks or events that the user needs to do
FBS approach (Lin et al. [26])	- Modeled the system behavior - Produced an explicit representation of failures and redundant functions	- Focused only on modeling particular aspects, such as the user’s role, values and needs. - The hardware platform was not modeled
EID approach (Lin et al. [26])	Interface the system design	- the display of information can distract the operator - Overlapping between different levels of information - No time consideration

## Data Availability

Not applicable.
